# Supplementary material for: Comprehensive Analysis of the Effect of 20(R)-Ginsenoside Rg3 on Stroke Recovery in Rats via the Integrative miRNA–mRNA Regulatory Network
Source: Molecules. 2022 Feb 27;27(5):1573. doi: 10.3390/molecules27051573 (PMC8911624; doi:10.3390/molecules27051573)
Supplement: Supplementary file 1 [file molecules-27-01573-s001.zip › molecules-1598375-supplementary.pdf]

Supplementary Materials

Comprehensive Analysis of the Effect of 20(*R*)-Ginsenoside Rg3 on Stroke Recovery in Rats via the Integrative miRNA–mRNA Regulatory Network

Rui Zhang [1,†], De-Yun Chen [2,†], Xing-Wei Luo [1], Yuan Yang [1], Xiao-Chao Zhang [1], Ren-Hua Yang [1], Peng Chen [1,*], Zhi-Qiang Shen [1,*] and Bo He [1,*]

[1] School of Pharmaceutical Sciences and Yunnan Key Laboratory of Pharmacology for Natural Products, Kunming Medical University, Kunming 650500, China; dtyz395zr@sina.cn (R.Z.); xingweiluo@aliyun.com (X.L.); yangyuanmail2@163.com (Y.Y.); 15887818479@139.com (X.-C.Z.); yangrenhua@kmmu.edu.cn (R.-H.Y.)

[2] Faculty of Food, Drugs and Health, Yunnan Vocational and Technical College of Agriculture, Kunming 650212, China; deyunchen@aliyun.com

* Correspondance: chenpeng@kmmu.edu.cn (P.C.); shzhq21cn@aliyun.com (Z.-Q.S.); hebo@kmmu.edu.cn (B.H)

† These authors contributed equally to this work.

Table S1. The primers were used for real-time quantitative PCR.

miRNA name	Primer sequence	Base number
rno-miR-19a-3p	TGTGCAAATCTATGCAAAACTGA	23
rno-miR-147	GTGTGCGGAAATGCTTCTGCTA	22
rno-miR-674-3p	CACAGCTCCCATCTCAGAACAA	22
rno-miR-671	TCCGGTTCTCAGGGCTCCACC	21
rno-miR-21a-5p	TAGCTTATCAGACTGATGTTGA	22
rno-miR-130b-5p	ACTCTTTCCCTGTTGCACTACT	22
U6	TGACACGCAAATTCGTGAAGCGT	23
Gfap	F: GATGTAGGAGTGGGTAGGGC	20
	R: CCCTCTCCGCATCCATACTT	20
Tgfb1	F: CCCTACATTTGGAGCCTGGA	20
	R: CGCACGATCATGTTGGACAA	20
Cd44	F: GCTCTGATTCTTGCCGTCTG	20
	R: CGGAGTCTCTGTTGGTTCCT	20
Pfkfb3	F: CAACTCCCCAACGGTGATTG	20
	R: TTTTCGGACTCTCATGGCCT	20
Serping1	F: AACGAGTCCTTCATCCAGCA	20
	R: AGGCGTGGTAGAGTTTCACA	20
C1qb	F: ACTACTTCACCTACCACGCC	20
	R: AGCTTCAAGACTACCCCACC	20
GAPDH	F: GGCACAGTCAAGGCTGAGAATG	22
	R: ATGGTGGTGAAGACGCCAGTA	21

Table S2. Differentially expressed mRNAs associated with the effect of 20(*R*)-ginsenoside Rg3 on stroke recovery.

Transcript_ID	Symbol	Sham	Model	Rg3	log2FoldChange		Pvalue	
					Sham_vs_Model	Model_vs_Rg3	Sham_vs_Model	Model_vs_Rg3
ENSRNOG00000000017	Steap1	0.429068	1.784127	0.224535	-2.421794094	3.072571238	0.009181827	0.000991443
ENSRNOG00000000137	Ly86	1.232169	5.039591	2.132352	-2.378597919	1.315758531	4.57E-13	1.43E-05
ENSRNOG00000000158	Cdo1	26.84255	76.34528	29.75808	-1.864560565	1.447373263	1.45E-05	0.000606417
ENSRNOG00000000187	Csf2rb	0.313088	15.30918	0.530901	-5.97767412	4.946077342	3.19E-13	1.43E-10
ENSRNOG00000000239	Ccl7	0	35.7736	0.142486	-14.27900972	7.178743827	5.88E-11	6.01E-07
ENSRNOG00000000394	Srgn	4.546667	30.31629	7.68173	-3.114438999	2.073277924	1.64E-07	0.000553965
ENSRNOG00000000655	Ptprc	1.073367	3.003609	1.20189	-1.764179073	1.394332018	1.67E-05	0.001046253
ENSRNOG00000000902	Hsph1	82.80933	163.2173	76.37713	-1.326273169	1.173658635	3.48E-06	3.26E-05
ENSRNOG00000000907	Alox5ap	1.197832	8.438242	3.256285	-3.175981673	1.458969505	4.68E-11	0.000475038
ENSRNOG00000000991	Arpc1b	5.052203	61.66863	13.94233	-3.968630844	2.236866789	2.76E-17	2.76E-07
ENSRNOG00000001006	Nptx2	23.31372	138.2795	17.50688	-2.950604009	3.087976406	7.92E-05	3.54E-05
ENSRNOG00000001149	Pxn	4.822803	14.93721	6.10581	-1.993397442	1.375072189	4.09E-07	0.000401535
ENSRNOG00000001193	Hsf2bp	0.102836	1.387968	0.175755	-4.093369164	3.347331829	1.53E-06	6.74E-05
ENSRNOG00000001224	Itgb2	1.861968	11.77823	2.269444	-3.021607426	2.469642893	1.16E-10	5.11E-08
ENSRNOG00000001309	Camkk2	48.34836	15.30151	42.80773	1.347380101	-1.436165602	0.000555595	0.00011442
ENSRNOG00000001414	Serpine1	0.242596	54.39652	0.415769	-8.162878645	7.135604143	1.63E-10	5.46E-09
ENSRNOG00000001607	Adamts1	2.361868	15.74226	1.365513	-3.104534847	3.627522086	5.34E-05	3.19E-06
ENSRNOG00000001704	Runx1	0.342472	1.757087	0.70417	-2.661493433	1.461427969	3.09E-08	0.000308833
ENSRNOG00000001794	Muc13	0	0.111326	0	-9.388811743	9.546790475	0.000338951	0.000201012
ENSRNOG00000001959	Mx1	0.307502	3.211327	0.478041	-3.746093536	2.832536073	1.84E-08	1.90E-05
ENSRNOG00000002045	Anxa3	8.823098	32.55113	15.53368	-2.231228325	1.152321121	2.93E-12	0.00015807
ENSRNOG00000002159	Gpat3	1.703766	6.9808	1.311162	-2.403605532	2.513484337	6.88E-05	2.91E-05
ENSRNOG00000002164	Nmu	0.284093	6.758432	0.354774	-4.93515767	4.380307349	0.001375005	2.94E-08
ENSRNOG00000002187	Ropn1	0.097778	0	0.027781	10.19558773	-8.807737952	2.79E-05	0.000451122

ENSRNOG00000002312	Atp10d	1.644597	3.111557	1.176593	-1.335203553	1.486828774	0.003056475	0.000893982
ENSRNOG00000002434	Tmem100	6.580228	11.49184	7.646341	-1.865333527	1.625226761	0.000131046	0.000492063
ENSRNOG00000002460	Serpinb2	0.038321	1.708931	0.004773	-5.810317005	8.481129691	0.003783418	8.89E-06
ENSRNOG00000002520	Litaf	12.23913	29.95299	8.975808	-1.655353894	1.831479729	0.000105191	1.82E-05
ENSRNOG00000002525	Ptgs2	6.95273	21.82997	5.805893	-2.014473587	2.009948051	0.001142879	0.000989115
ENSRNOG00000002657	Pla2g4a	1.824737	4.575885	1.832147	-1.680540877	1.405382658	4.45E-05	0.000393279
ENSRNOG00000002771	Ereg	0	0.255882	0	-10.98935499	11.14749014	0.000331395	0.000192558
ENSRNOG00000002792	Cxcl2	0	7.126827	0.022406	-12.88632689	8.174397199	6.20E-08	7.53E-06
ENSRNOG00000002802	Cxcl1	0.01265	8.096651	0.128136	-9.517208495	6.02280512	1.24E-06	4.93E-05
ENSRNOG00000002810	Gfpt2	0.895272	4.843365	1.69201	-2.789596777	1.789155144	5.41E-08	0.00012034
ENSRNOG00000002919	Gfap	151.1962	1523.055	283.4966	-3.709986039	2.501480198	3.78E-28	1.71E-09
ENSRNOG00000002926	Uap1	9.56001	42.24005	9.161844	-2.506055621	2.300293439	1.62E-05	6.32E-05
ENSRNOG00000002946	Socs3	0.686878	36.23868	1.159759	-6.076203366	5.055520284	7.30E-07	7.97E-07
ENSRNOG00000003069	Cd38	4.223536	8.158848	3.560554	-1.298822385	1.273086827	0.000165627	5.24E-05
ENSRNOG00000003088	Arhgap31	4.986322	10.83671	4.462438	-1.477884158	1.368621512	0.000257152	0.000769081
ENSRNOG00000003289	Lap3	19.96464	40.24178	20.8051	-1.361540647	1.032499884	1.09E-05	0.000601343
ENSRNOG00000003546	Tnfrsf12a	1.54574	36.12768	1.197726	-4.901307629	4.993674518	6.61E-07	2.81E-07
ENSRNOG00000003703	Mcm6	1.594028	7.224993	2.096649	-2.535364539	1.866987657	1.46E-08	4.43E-06
ENSRNOG00000003745	Atf3	0.72741	16.85721	0.233215	-4.419890893	6.086742572	3.27E-05	6.72E-10
ENSRNOG00000003809	Sat1	39.73848	102.7199	46.82588	-1.727982147	1.217847361	4.74E-06	0.00051322
ENSRNOG00000003895	Rgs1	0.716144	8.475764	0.334065	-3.92247434	4.752415674	0.000130394	6.23E-06
ENSRNOG00000003927	Cd55	0.651528	4.886055	0.723666	-3.265562477	2.843741874	3.27E-05	0.000176783
ENSRNOG00000003975	Pfn1	64.08555	256.3443	127.5924	-2.352629012	1.092096839	3.86E-11	0.000995562
ENSRNOG00000004111	Soat1	5.775607	12.4517	5.585765	-1.458191218	1.2398918	5.15E-05	0.000412348
ENSRNOG00000004117	Kcnv1	29.49323	7.705843	20.74791	1.610592887	-1.36940233	5.95E-05	0.000505845
ENSRNOG00000004179	Nts	4.67693	15.0094	3.278222	-2.054767379	2.275075361	0.003053021	0.000149165
ENSRNOG00000004380	Il12b	0	0.36003	0	-10.79560309	10.95372825	1.33E-05	5.94E-06
ENSRNOG00000004500	Myc	0.64714	12.93399	2.031631	-5.33926615	2.997005046	1.79E-08	0.000124728
ENSRNOG00000004603	Srgap1	2.418244	5.52681	2.461999	-1.550273017	1.250331376	2.65E-05	0.000604501
ENSRNOG00000004649	Il1b	0.039773	3.09928	0.243444	-6.622528751	3.756607928	2.64E-05	0.000818316
ENSRNOG00000004883	Erh	18.41192	13.83161	6.463222	-1.145737973	1.309542091	0.009077511	0.000794817
ENSRNOG00000005206	Kcnq3	38.44308	9.708984	18.55369	1.549992827	-1.30330194	3.84E-07	0.000747666
ENSRNOG00000005214	Plek	3.204843	16.29563	7.059565	-2.577473144	1.230854126	1.73E-14	0.000196964

ENSRNOG00000005424	Odc1	21.14244	68.10707	22.66417	-1.947329475	1.653626721	4.02E-05	0.000357256
ENSRNOG00000005620	Lcp2	1.051708	4.035488	1.545097	-2.291400144	1.470253475	1.02E-07	0.000274299
ENSRNOG00000005731	Birc3	0.25099	2.971655	0.263571	-3.921906921	3.590703179	3.55E-06	1.01E-05
ENSRNOG00000005809	Arhgdib	5.427036	28.60651	11.6006	-2.744143721	1.380964639	1.94E-17	1.34E-05
ENSRNOG00000005825	Lyz2	34.46258	143.2088	56.46497	-2.414922176	1.436972384	1.71E-08	0.001029746
ENSRNOG00000005871	Il1rn	0.19537	11.60404	0.275527	-6.251642261	5.491539521	2.24E-08	3.80E-08
ENSRNOG00000005935	A3galt2	1.435943	13.25028	1.832479	-3.573760823	2.951541939	1.51E-07	5.01E-06
ENSRNOG00000006094	Cd44	2.305289	47.48718	3.778554	-4.39689286	3.51870942	9.98E-13	4.00E-09
ENSRNOG00000006320	Ptges	0.466412	15.59912	1.375214	-5.422762173	3.605174266	1.59E-10	7.63E-06
ENSRNOG00000006778	Mmp19	0.225899	6.895472	1.660327	-5.278495438	2.143735814	1.18E-10	0.001034349
ENSRNOG00000006828	Baz1a	0.926814	2.95615	0.78835	-2.039058598	1.997675595	0.000143187	0.000116239
ENSRNOG00000006921	Rbl1	0.909115	1.707285	0.62272	-1.255631823	1.527991758	0.005198757	0.000652937
ENSRNOG00000006932	AABR07029272.1	1.211117	3.249365	1.507332	-1.765504569	1.186775573	1.61E-07	0.000197802
ENSRNOG00000006940	Ncf4	0.181209	10.35197	0.834577	-6.202792097	3.73154638	9.32E-16	1.80E-09
ENSRNOG00000007002	Lif	0.104917	6.312887	0.09306	-5.625536967	5.544946454	7.75E-06	5.29E-06
ENSRNOG00000007060	Plin2	2.571133	43.93683	3.480342	-4.46288371	3.759296489	1.07E-11	3.86E-09
ENSRNOG00000007062	Rin3	0.699158	4.531037	1.827067	-3.034767089	1.391063369	6.42E-16	0.000128964
ENSRNOG00000007081	Xdh	1.041399	2.839296	0.664442	-1.805765479	2.189180878	0.00035234	2.07E-05
ENSRNOG00000007089	Lgmn	26.94112	90.45934	36.96527	-2.103414782	1.378594723	8.97E-10	4.22E-05
ENSRNOG00000007142	B3gat1	41.68238	13.31428	30.67071	1.327413642	-1.150254193	0.000141203	0.000858245
ENSRNOG00000007159	Ccl2	0.070712	108.8863	0.256009	-10.91025716	8.832963281	6.51E-08	8.82E-10
ENSRNOG00000007281	Flnc	3.225038	19.2149	3.955228	-2.954761318	2.387231403	1.88E-08	4.69E-06
ENSRNOG00000007302	Fbn1	1.187807	9.028156	1.734139	-3.282625853	2.47088051	2.10E-08	2.27E-05
ENSRNOG00000007338	Fbln2	4.173384	9.354309	2.564034	-1.535255671	1.957739996	0.006200806	0.000417984
ENSRNOG00000007350	Rac2	1.216867	13.81997	3.497611	-3.862171631	2.069088378	9.06E-14	1.39E-06
ENSRNOG00000007390	Nfkbia	14.66626	55.74839	12.14227	-2.282722482	2.292788783	1.29E-05	5.52E-06
ENSRNOG00000007457	Serping1	1.387886	49.53224	18.28385	-5.500408439	1.522308571	1.89E-27	0.000235617
ENSRNOG00000007545	Angptl4	3.525753	16.19055	2.68071	-2.559834383	2.686313162	2.24E-05	6.24E-06
ENSRNOG00000007584	Ehd4	5.410751	13.53845	5.484175	-1.676091901	1.392329412	1.81E-05	0.000174187
ENSRNOG00000007650	Cd63	34.33716	277.7813	84.71673	-3.380736368	1.803296748	3.42E-16	3.71E-06
ENSRNOG00000007679	Cyth4	4.162606	10.77297	4.903011	-1.734608292	1.21721771	7.50E-06	0.000913094
ENSRNOG00000007682	Gria3	80.43405	16.27382	36.3314	1.983002697	-1.103288743	1.07E-10	0.000262111
ENSRNOG00000007918	Tbxas1	0.661158	4.196023	1.254406	-3.02016419	1.829301501	1.16E-16	2.53E-07

ENSRNOG00000008015	Fos	7.620998	68.66133	5.52085	-3.548598773	3.736500525	5.22E-05	2.02E-05
ENSRNOG00000008180	Lyn	4.745708	13.11201	5.011217	-1.817862117	1.471531959	8.79E-09	2.07E-06
ENSRNOG00000008182	Htra3	2.261168	13.48312	6.255749	-2.925768134	1.189277151	4.67E-14	0.000533445
ENSRNOG00000008215	Trim47	2.240002	12.48199	1.681511	-2.838874188	2.985894027	8.39E-05	2.88E-05
ENSRNOG00000008301	Tagln2	10.23588	78.70447	18.44895	-3.305006794	2.186714777	8.54E-12	2.99E-06
ENSRNOG00000008409	Myo1f	0.916019	5.722006	1.652493	-2.998379529	1.878709199	1.59E-12	3.11E-06
ENSRNOG00000008412	Gprc5a	0.25088	3.189009	0.159437	-4.024280483	4.423155936	0.000105738	1.23E-05
ENSRNOG00000008676	Emp1	4.166631	24.96561	6.963922	-3.094079541	2.031687474	2.09E-06	0.00066642
ENSRNOG00000008816	Gpnmb	2.864476	56.9058	8.198007	-4.679720052	2.89767592	1.52E-20	1.18E-08
ENSRNOG00000009005	Slco2a1	0.297874	5.647548	2.450181	-4.566798032	1.310751966	2.60E-18	0.000152914
ENSRNOG00000009088	Txnrd1	17.62083	38.81866	17.23063	-1.494945707	1.260935854	4.27E-05	0.000510442
ENSRNOG00000009331	Hck	0.653909	9.239087	1.588352	-4.185742535	2.634171847	3.29E-16	1.93E-09
ENSRNOG00000009740	Slco1c1	22.60661	6.439062	15.67991	1.485176302	-1.230283666	9.28E-06	0.000312821
ENSRNOG00000009771	Cdh10	18.7227	4.778206	10.1486	1.64906922	-1.034900512	2.07E-07	0.001028614
ENSRNOG00000009785	Cdkn3	0.700305	3.440505	0.606781	-2.666992743	2.60198622	0.001301698	0.000844173
ENSRNOG00000009912	Fgr	0.528973	6.681776	0.681878	-4.025991567	3.391811466	4.82E-08	1.34E-06
ENSRNOG00000010105	S100a11	1.367061	45.57683	8.828029	-5.398309958	2.466205598	2.84E-11	0.000180178
ENSRNOG00000010107	AABR07025295.1	3.222299	6.562578	3.005026	-1.382556358	1.215558162	0.000168119	0.000854321
ENSRNOG00000010165	Tnfaip2	2.027178	11.14641	1.4248	-2.822965854	3.054055798	1.66E-05	8.69E-06
ENSRNOG00000010208	Timp1	0.957506	442.1998	22.08231	-9.209321788	4.437456684	2.63E-25	8.40E-08
ENSRNOG00000010278	Il6	0.213909	7.031325	0.029027	-5.387772259	7.962320423	2.69E-05	9.99E-07
ENSRNOG00000010319	Lcp1	4.841377	30.33306	5.393326	-3.010940452	2.58313625	1.25E-12	3.22E-10
ENSRNOG00000010331	Ctsb	130.243	409.2003	194.1616	-2.003970147	1.161630104	5.13E-10	0.000311332
ENSRNOG00000010362	Anxa2	4.372696	119.0204	31.10409	-5.118833407	2.028998031	1.79E-19	2.39E-05
ENSRNOG00000010448	Ptbp1	8.563962	33.13495	12.98766	-2.423685969	1.468651178	8.11E-08	0.000370532
ENSRNOG00000010478	Serpina3n	0	124.5057	1.449908	-19.14921542	6.55770923	9.15E-30	4.72E-08
ENSRNOG00000010513	Tfpi2	0.173038	5.593955	0.320218	-5.37308354	4.217975264	9.25E-06	0.000149026
ENSRNOG00000010524	Cryab	10.71984	60.71192	23.27184	-2.859113976	1.473991985	3.71E-09	0.000696523
ENSRNOG00000010549	Tspo	2.127155	39.45998	4.851274	-2.744631298	2.258652041	5.65E-08	2.19E-06
ENSRNOG00000010555	LOC108348161	55.84243	13.52031	72.65161	1.717192319	-2.363636205	1.01E-05	5.99E-10
ENSRNOG00000010584	Tmem123	4.226847	7.492365	3.242025	-1.202731825	1.29144676	0.001697327	0.000538068
ENSRNOG00000010645	Lgals3	0.71145	75.84601	5.252358	-7.084505909	3.954531273	7.60E-18	3.94E-09
ENSRNOG00000010747	Dap	2.673539	19.53538	9.674391	-3.221256602	1.098555311	1.63E-18	0.000948885

ENSRNOG00000010797	Esm1	0.034277	3.932665	0.229977	-7.131232503	4.192993762	3.92E-06	0.000103441
ENSRNOG00000010799	Noct	11.06685	22.52612	8.776729	-1.387490213	1.452909559	0.001405334	0.000773083
ENSRNOG00000010833	Mthfd2	1.596542	7.983449	1.958006	-2.680246374	2.117746088	6.17E-06	0.000135033
ENSRNOG00000010897	Nek6	12.75959	29.43223	11.11635	-1.562626621	1.490913387	0.000261606	0.000305565
ENSRNOG00000010994	Has1	0.057518	1.617969	0.11537	-5.144092305	3.910339548	0.001197384	0.000913552
ENSRNOG00000011039	Gch1	0.712278	4.05706	0.412075	-2.878514681	3.40043158	2.53E-05	4.34E-06
ENSRNOG00000011068	Papss2	4.230072	7.832425	2.956418	-1.22157368	1.548468788	0.007986291	0.00059002
ENSRNOG00000011205	Ccl3	0.721261	6.81109	0.667256	-4.273852179	3.753491252	0.000608101	0.001069717
ENSRNOG00000011250	Inmt	0.24326	88.48914	4.992303	-8.845004798	4.252233583	3.25E-16	1.31E-06
ENSRNOG00000011316	Fam167a	0.210575	1.12193	0.153201	-2.779048796	2.962257925	0.000951666	0.000527476
ENSRNOG00000011346	Ehd2	2.458701	15.72391	4.370507	-3.033773962	1.936485949	2.71E-09	5.08E-05
ENSRNOG00000011406	Ccl4	0.163353	6.445362	0.298289	-5.620069895	4.491261726	0.000408269	0.000250172
ENSRNOG00000011459	Rhbdf2	0.106836	2.229352	0.44579	-4.746153639	2.410559491	1.28E-08	9.68E-05
ENSRNOG00000011559	Cnn3	34.61831	116.9159	53.90931	-2.111758139	1.202309878	3.38E-10	0.000202022
ENSRNOG00000011668	Nfil3	5.234342	17.47091	2.806244	-2.101020289	2.732981254	0.000442229	7.21E-06
ENSRNOG00000011774	Fblim1	0.089056	2.925955	0.474664	-5.324120402	2.72420381	5.37E-09	0.000294279
ENSRNOG00000011821	S100a4	1.784038	38.8114	4.727391	-4.815806945	3.139850588	8.07E-10	4.69E-06
ENSRNOG00000011824	Trh	1.085563	50.22171	2.851163	-5.909052236	4.244632108	4.33E-07	2.54E-05
ENSRNOG00000011913	Cp	2.973888	39.26166	9.964166	-4.100838736	2.092005817	1.53E-17	3.23E-05
ENSRNOG00000012049	Sox7	0.262203	2.398926	0.148198	-3.55182653	4.108570029	0.00143678	0.000246734
ENSRNOG00000012094	Ltbp2	0.192593	1.272918	0.225508	-3.029990199	2.575867054	5.78E-05	0.00010129
ENSRNOG00000012109	Otulinl	2.127331	7.46352	2.224649	-2.166852337	1.836529972	1.07E-05	0.000109215
ENSRNOG00000012172	Spi1	1.27039	9.155996	2.555433	-3.207887123	1.931390647	1.06E-09	3.58E-05
ENSRNOG00000012228	Skap2	4.270305	9.968496	4.742764	-1.568106457	1.144117585	7.37E-09	7.41E-06
ENSRNOG00000012280	Ptx3	0.369856	24.71193	0.297404	-7.173918861	6.990866625	1.33E-07	4.30E-08
ENSRNOG00000012543	Mcm3	0.62015	4.462034	0.657252	-3.200008201	2.850913049	3.18E-08	3.47E-07
ENSRNOG00000012582	Eif4ebp1	2.380309	24.16636	9.404328	-3.699795656	1.453140588	1.52E-15	0.000517078
ENSRNOG00000012630	Rhoc	6.924728	54.3734	15.01873	-3.412441017	2.027915799	3.82E-08	0.000168215
ENSRNOG00000012698	Chsy1	7.814113	16.35325	6.661317	-1.423318948	1.382919139	0.000213044	0.000322353
ENSRNOG00000012749	C1qb	11.52996	122.3901	42.87784	-3.756858752	1.596572335	2.51E-25	5.67E-06
ENSRNOG00000012779	Msr1	0.042674	2.929867	0.174641	-6.448591516	4.167324203	7.50E-11	2.98E-08
ENSRNOG00000012804	C1qc	12.61939	103.5194	34.10633	-3.387080978	1.686227081	4.36E-17	1.63E-05
ENSRNOG00000012843	Aspg	0.083593	5.587449	0.896792	-6.416316077	2.735171109	6.27E-12	0.00012211

ENSRNOG00000012886	Maff	0.857058	13.66811	0.558044	-4.36197423	4.72034675	7.78E-07	2.54E-07
ENSRNOG00000012956	Tgm2	3.53073	18.04361	3.886497	-2.710736675	2.309549695	1.72E-07	7.73E-06
ENSRNOG00000013014	Cyba	1.759723	35.09187	11.85053	-4.667521294	1.651443991	7.24E-23	2.27E-05
ENSRNOG00000013090	Gadd45g	3.910041	66.75934	4.336882	-4.455249112	4.045579144	6.80E-07	1.37E-06
ENSRNOG00000013170	LOC100360218	1.373065	8.100056	2.32787	-2.917463729	1.892326838	1.59E-09	3.27E-05
ENSRNOG00000013190	Rnaset2	9.142836	45.79355	22.30657	-2.675514516	1.118832801	1.48E-15	0.000236886
ENSRNOG00000013220	Arhgap45	1.464851	4.230634	1.799549	-1.883183851	1.316570343	7.82E-08	0.000126551
ENSRNOG00000013250	NEWGENE_1310561	0.456579	0	3.393843	9.961139022	-13.29342823	0.009699323	0.000343185
ENSRNOG00000013526	Rassf4	1.646903	6.748115	1.77122	-2.392897502	2.018101386	3.40E-07	6.56E-06
ENSRNOG00000013588	Glra1	0.259575	0.886326	0.117941	-2.132093834	2.986341319	0.003677116	0.001065985
ENSRNOG00000013668	Capg	1.357342	21.32273	2.024007	-4.335366104	3.495588737	4.69E-13	1.73E-09
ENSRNOG00000013747	Sh3bp2	1.079681	4.955073	1.796216	-2.554317792	1.554376715	3.05E-07	0.000600938
ENSRNOG00000013791	Enpp3	0.253197	5.581477	1.104141	-4.814850167	2.429433182	3.50E-14	5.63E-06
ENSRNOG00000013794	Rbp1	2.467465	55.62567	23.09245	-4.785110335	1.355481817	2.85E-23	0.000637519
ENSRNOG00000013859	Bpifa1	0.036514	3.421855	0.294675	-6.793525603	3.563256993	0.000195973	0.000859566
ENSRNOG00000013948	Zc3hav1	1.649536	6.049909	2.016408	-2.227150281	1.667694356	4.18E-08	5.25E-05
ENSRNOG00000013967	Blnk	1.560178	7.591719	3.585827	-2.619738379	1.156003978	7.80E-15	0.000102489
ENSRNOG00000013973	Lcn2	0.283562	59.69008	2.729175	-8.061928025	4.564053642	1.78E-23	2.29E-08
ENSRNOG00000013987	Sbno2	2.6556	11.64106	2.555928	-2.652411255	2.290926775	4.96E-08	1.70E-06
ENSRNOG00000014061	Dusp5	1.152101	5.797188	1.030401	-2.699340063	2.592017153	0.000396712	0.000477125
ENSRNOG00000014117	Hmox1	2.504441	244.3849	8.3048	-6.973823701	4.988860807	3.05E-17	1.70E-09
ENSRNOG00000014165	Ssr1	14.43412	33.64343	15.9042	-1.460507648	1.478187163	9.75E-05	4.80E-05
ENSRNOG00000014241	Ece1	7.317701	18.05024	8.397708	-1.657788317	1.191560865	4.07E-06	0.00066681
ENSRNOG00000014276	Plce1	1.086783	2.453458	0.889123	-1.564566664	1.583038647	4.78E-05	2.13E-05
ENSRNOG00000014294	Ptpn6	1.730136	14.23606	4.145036	-3.389002583	1.861891253	2.04E-07	0.000916016
ENSRNOG00000014320	Inhba	2.631228	14.62491	2.527172	-2.967592051	2.700424188	3.29E-05	0.00012636
ENSRNOG00000014327	Csrp3	0	0.465754	0	-10.65460785	10.81272151	1.12E-05	4.96E-06
ENSRNOG00000014336	Mcm5	0.894161	4.22021	0.490172	-2.582260699	3.187226462	4.59E-08	3.61E-09
ENSRNOG00000014350	Ccn1	2.093177	14.07514	1.290352	-3.139083048	3.547900562	0.008668865	0.000772526
ENSRNOG00000014378	Il1r2	0.052713	3.078105	0.069558	-6.226132866	5.565579247	1.65E-08	0.000277287
ENSRNOG00000014398	Scara5	0.253086	4.317555	0.409624	-4.451578893	3.489514504	3.03E-12	7.16E-09
ENSRNOG00000014426	Lox	0.937802	16.08489	1.740624	-4.452425572	3.304050743	7.48E-08	4.95E-05
ENSRNOG00000014524	S1pr3	1.742078	11.71233	1.089407	-3.118397587	3.52448061	1.09E-05	8.89E-07

ENSRNOG00000014532	Lbp	0.024375	43.32438	2.209903	-11.11605418	4.403203653	2.54E-12	5.77E-06
ENSRNOG00000014741	Kcnu1	0	0.013294	0	-9.184551672	9.342503531	0.0004366	0.000263547
ENSRNOG00000014751	Ret	1.344932	4.952366	1.095742	-2.236395759	2.267158249	3.72E-06	5.52E-06
ENSRNOG00000014797	Tmbim1	11.13309	72.14071	9.654638	-3.022683437	3.035775035	3.23E-07	1.67E-07
ENSRNOG00000014838	Glipr2	0.658906	11.38545	1.418055	-4.486047649	3.099276842	1.72E-10	9.08E-07
ENSRNOG00000014840	Gna14	1.937986	0.321106	0.983842	2.272800945	-1.556592285	5.61E-08	0.000927573
ENSRNOG00000014872	Sec24d	2.965746	8.991937	3.766852	-1.958956528	1.342074913	1.34E-07	0.000171153
ENSRNOG00000014956	Slc11a1	1.333819	7.588776	1.379132	-2.953595112	2.673686801	3.25E-05	6.25E-05
ENSRNOG00000014961	Pdpn	3.411935	64.2425	11.98158	-4.594562033	2.519217317	4.27E-16	5.12E-07
ENSRNOG00000015055	Scg2	37.33852	117.0663	41.08537	-2.013371448	1.602645381	3.33E-05	0.000601531
ENSRNOG00000015078	Ifitm3	3.630484	108.2375	13.39761	-5.255830788	3.11141165	1.65E-13	1.06E-06
ENSRNOG00000015156	Gal	0.489156	14.80557	1.548302	-5.297375983	3.351238743	7.13E-07	9.28E-05
ENSRNOG00000015160	Gem	1.219949	15.9176	0.615492	-4.070841641	4.788201865	5.67E-05	2.69E-06
ENSRNOG00000015278	Myl12a	14.92863	83.43625	32.29005	-2.84114426	1.45879764	3.33E-12	0.0001918
ENSRNOG00000015441	Il4r	2.447049	8.893525	1.523419	-2.220964108	2.646367027	4.12E-05	1.63E-06
ENSRNOG00000015478	Aicda	0	0.120682	0	-9.038612902	9.196525879	0.000852289	0.000556718
ENSRNOG00000015496	Tpm4	7.392628	57.9292	13.74022	-3.344534518	2.178001528	1.68E-11	6.60E-06
ENSRNOG00000015727	Loxl4	0.247052	2.888605	0.382691	-3.911190492	3.009373399	4.21E-05	0.00074334
ENSRNOG00000015845	Fam129b	14.75817	57.44456	17.26795	-2.32362855	1.828101823	6.03E-07	7.92E-05
ENSRNOG00000015894	Dock8	2.164904	5.007917	2.431852	-1.677815598	1.23875603	2.34E-06	0.000221461
ENSRNOG00000015992	Ccl20	0.01375	9.012569	0.012082	-9.556412843	9.475801448	8.38E-05	9.19E-05
ENSRNOG00000016166	Pdlim1	1.744295	8.355617	2.418079	-2.617839071	1.88193365	3.56E-08	3.05E-05
ENSRNOG00000016257	Cotl1	12.54993	51.61692	22.88485	-2.389236371	1.255289218	7.56E-16	8.96E-06
ENSRNOG00000016281	Col4a1	5.095123	12.41868	2.684978	-1.653584878	2.306587705	0.001195929	1.27E-05
ENSRNOG00000016316	Mcm2	1.157679	6.05795	1.709846	-2.740703271	1.910757186	6.72E-07	0.000164913
ENSRNOG00000016413	Pstpip1	0.675013	4.29719	1.197978	-3.028546178	1.937343688	5.13E-08	9.10E-05
ENSRNOG00000016496	Ctsc	2.16876	17.26808	3.589933	-3.347634099	2.3544812	9.45E-13	2.86E-07
ENSRNOG00000016535	Ccl22	0	0.20827	0	-9.591469888	9.749474796	0.000331569	0.000188732
ENSRNOG00000016831	Serpinh1	13.60324	84.21868	32.02923	-3.023162795	1.480410306	2.08E-10	0.000718042
ENSRNOG00000017194	Prdx1	79.86875	161.8462	79.05539	-1.375556088	1.11626123	1.76E-05	0.000191662
ENSRNOG00000017311	Me3	15.22957	3.758803	18.90011	1.725045725	-2.307977513	0.006003995	0.000257235
ENSRNOG00000017386	Il11	0.053704	11.94855	0.072922	-8.109058832	7.444074535	3.27E-06	1.71E-05
ENSRNOG00000017410	Loxhd1	0.00719	0.142571	0.015874	-4.650450033	3.263300678	0.001301143	0.000698472

ENSRNOG00000017453	Ptpn2	4.541022	9.684762	4.116393	-1.45006618	1.323199935	0.000300755	0.00083132
ENSRNOG00000017469	Anxa1	3.192162	26.19711	7.047588	-3.387708324	1.982743225	4.01E-10	6.93E-05
ENSRNOG00000017686	Pi15	0.018271	1.252546	0.034408	-6.472371833	5.268443923	0.000842326	0.00077142
ENSRNOG00000017819	Cd14	0.260558	37.32254	0.983262	-7.477005064	5.341864366	2.50E-11	5.72E-08
ENSRNOG00000017869	Irf8	2.105355	8.616895	3.006827	-2.387899517	1.599013694	5.85E-08	3.73E-05
ENSRNOG00000017874	Cd53	3.876073	12.62908	4.85629	-2.065306142	1.463689153	1.65E-07	0.000122712
ENSRNOG00000017980	Itgal	0.372143	2.388393	0.352797	-3.040257734	2.847080639	4.24E-07	3.03E-07
ENSRNOG00000018087	Vim	23.4748	299.7038	133.4181	-4.021356174	1.252668916	1.10E-27	0.000277707
ENSRNOG00000018109	Clic4	42.37265	105.2371	43.79432	-1.473275152	1.371315121	4.07E-05	0.000130551
ENSRNOG00000018371	Tubb6	1.457564	57.27127	3.944251	-5.663067669	3.959301914	5.59E-14	9.55E-10
ENSRNOG00000018450	LOC100911440	24.08337	0	21.43169	15.82222613	-16.11952811	7.33E-09	5.51E-09
ENSRNOG00000018646	Hbegf	2.419372	20.8403	1.747288	-3.46691205	3.673029014	2.98E-06	4.81E-07
ENSRNOG00000018659	Csf1	2.555552	10.76107	3.138856	-2.363713032	1.859905674	6.94E-07	4.18E-05
ENSRNOG00000018681	Nes	0.649706	5.562679	0.727707	-3.437450266	3.031940718	5.78E-05	0.000191338
ENSRNOG00000018752	Clcf1	0.247876	3.073192	0.132887	-3.977068455	4.620512463	0.000155145	2.61E-05
ENSRNOG00000018911	Pfkfb3	12.51025	30.83576	10.52771	-1.657780474	1.631291187	0.000211322	0.00023908
ENSRNOG00000019179	Ggta1	1.425621	4.986177	1.425783	-2.155050131	1.905250583	4.16E-05	0.00011212
ENSRNOG00000019202	PVR	1.12688	7.494073	1.259099	-3.097926456	2.667829146	3.96E-06	3.57E-05
ENSRNOG00000019203	Eya2	0.547107	12.07847	4.564573	-4.68331918	1.501831339	1.71E-23	0.00018042
ENSRNOG00000019229	Fbl	7.232945	36.09527	14.8841	-2.677564875	1.365905919	1.91E-09	0.000678665
ENSRNOG00000019293	AABR07006025.1	0	1.784952	0	-13.02909945	13.18729122	0.00051094	0.000385197
ENSRNOG00000019358	Esr1	0.220076	0.692301	0.162446	-3.259971065	2.946897172	2.99E-11	3.23E-10
ENSRNOG00000019387	Ifi30	4.212025	24.67623	8.646421	-2.903670069	1.597846954	4.78E-08	0.000943404
ENSRNOG00000019440	Kcnn4	0.213761	2.486953	0.356149	-3.935054736	2.962547649	1.96E-05	4.97E-05
ENSRNOG00000019549	Akap12	4.124033	20.0887	7.600875	-2.597759386	1.482887256	2.57E-08	0.000568114
ENSRNOG00000019556	Cd9	33.67803	81.33993	38.20927	-1.630692382	1.174914649	2.62E-06	0.000472653
ENSRNOG00000019661	Gdf15	0.10246	4.79898	0.109583	-5.827568167	5.480572075	0.000319825	6.12E-05
ENSRNOG00000019728	Itgad	3.507667	20.62849	3.023532	-2.921936571	2.862271595	3.21E-11	3.41E-10
ENSRNOG00000019742	Stat3	15.78748	53.31467	15.66129	-2.114522919	1.859714051	1.27E-07	2.99E-06
ENSRNOG00000019780	Sypl2	0.381365	4.075203	0.392672	-3.778901227	3.474520407	8.30E-06	3.00E-05
ENSRNOG00000019822	Gadd45b	2.82069	39.1759	5.378538	-4.105394552	2.93548338	2.77E-06	0.000213382
ENSRNOG00000019892	Lrrfip1	1.044725	3.907386	1.175998	-2.26378659	1.822215108	2.51E-06	6.23E-05
ENSRNOG00000020009	Npas4	1.83686	14.46334	1.673649	-3.350439011	3.210311812	0.000500232	0.000474366

ENSRNOG00000020136	Tgm1	0.049958	1.220027	0.034757	-4.979286598	5.219775504	0.00148578	3.87E-05
ENSRNOG00000020182	Mvp	2.526197	19.42303	5.352077	-3.300913984	1.950078872	3.69E-14	2.44E-06
ENSRNOG00000020298	Bag3	1.328142	28.45447	5.226681	-4.780886924	2.539919421	2.61E-19	5.15E-08
ENSRNOG00000020300	Lsp1	0.277078	21.89651	2.069675	-6.670072308	3.502171121	2.01E-06	2.14E-11
ENSRNOG00000020346	Best1	2.558022	26.35548	13.1448	-3.715448579	1.085496725	1.10E-22	0.001035233
ENSRNOG00000020465	Ripk3	0.266985	8.004988	0.531353	-5.266004074	4.011638599	9.83E-10	4.88E-07
ENSRNOG00000020478	Camk4	34.04025	12.39459	27.39618	1.135856832	-1.089200348	0.000358421	0.00020816
ENSRNOG00000020552	Fosl1	0.19638	8.41983	0.155086	-5.768514067	5.858485586	4.76E-06	1.03E-06
ENSRNOG00000020579	Col7a1	0.26835	1.399167	0.185122	-2.743905876	3.015455648	0.001081291	0.000330646
ENSRNOG00000020652	Tgfb1	2.475835	21.04701	5.635275	-3.450725031	1.99652615	2.55E-11	2.44E-05
ENSRNOG00000020657	Shc1	3.423477	23.30589	6.123304	-3.126707317	2.029050511	1.78E-08	0.000108662
ENSRNOG00000020679	Icam1	0.947553	9.018488	1.723042	-3.611966544	2.483423689	2.99E-07	0.000164114
ENSRNOG00000020684	Vat1	13.84345	37.8095	15.92669	-1.806819687	1.334057098	7.72E-06	0.000460993
ENSRNOG00000020843	Ftl1	204.3446	618.0615	307.2334	-2.039217598	1.092648303	4.21E-09	0.001078514
ENSRNOG00000020845	Tyrobp	10.02541	63.59024	16.45948	-3.020775119	2.036978599	9.83E-12	7.58E-07
ENSRNOG00000020991	Ms4a6a	0.502444	4.044795	0.751612	-3.365911368	2.51927329	3.85E-06	0.000616463
ENSRNOG00000021062	Fxyd5	1.14309	39.90162	12.99048	-5.147430543	1.649670841	2.66E-26	8.21E-05
ENSRNOG00000021161	Fermt3	1.407235	9.496798	2.240306	-3.107995937	2.17500796	4.30E-09	6.34E-06
ENSRNOG00000021199	Fcgr1a	0.789346	6.816176	2.102038	-3.433532026	1.789895329	2.71E-09	0.000356278
ENSRNOG00000021817	Irak2	2.888698	4.840281	1.762657	-1.096316259	1.540395902	0.009172555	0.000284358
ENSRNOG00000021833	Myrfl	0	0.010265	0	-8.788859674	8.946734097	0.000656276	0.00045371
ENSRNOG00000021856	Lat2	0.691034	4.360472	1.216191	-2.900983317	1.901546011	9.65E-07	0.000332473
ENSRNOG00000022859	Trem1	0.028746	3.209523	0.079661	-7.168391756	5.447664953	0.000631181	0.00072592
ENSRNOG00000022937	Bpifc	0	0.020499	0	-9.244569317	9.402532493	0.000226602	0.000134938
ENSRNOG00000023226	S100a10	14.08326	113.0228	30.51468	-3.363518275	1.981534573	2.22E-07	0.000956859
ENSRNOG00000023257	Adamts9	1.175631	2.819359	0.694291	-1.631122869	2.119385548	0.00225974	0.000124258
ENSRNOG00000023463	Parp9	1.393235	6.671471	2.233466	-2.613544068	1.663829955	1.33E-08	0.000158501
ENSRNOG00000023546	Hspb1	0.937441	384.8329	5.230747	-9.047648983	6.308343016	2.90E-25	5.84E-14
ENSRNOG00000023831	AABR07027258.1	0.675478	0.039255	0.64675	3.715908708	-3.850418089	0.000476524	0.000136549
ENSRNOG00000023991	Rab20	0.457218	3.536654	0.89007	-3.31248783	2.079975475	1.85E-07	0.000169152
ENSRNOG00000024028	Sprr1a	0.586123	16.47202	0.696689	-5.157329175	4.653316212	7.50E-06	1.42E-05
ENSRNOG00000024082	Gldn	1.262543	5.074	0.325522	-2.373498697	4.05629377	0.003142637	2.66E-06
ENSRNOG00000024115	C6	0.051758	3.947721	0.523637	-5.527877666	2.594390983	2.11E-10	0.000228282

ENSRNOG00000024159	Fcer1g	1.791748	41.3144	15.27227	-4.873690911	1.518984633	2.03E-21	0.000219351
ENSRNOG00000024382	Fcgr3a	0.710608	19.25576	2.006926	-5.064831912	3.348917983	4.03E-18	9.06E-11
ENSRNOG00000024661	Jpt2	3.76904	7.962443	3.226705	-1.57217783	1.39509023	0.003081592	0.001000749
ENSRNOG00000024882	Pax1	0	0.466103	0	-11.57236831	11.73052755	9.96E-05	5.55E-05
ENSRNOG00000025273	AABR07068728.1	1.492843	2.851868	1.239379	-1.554866817	1.548288554	0.001592518	0.000738653
ENSRNOG00000025679	Stk40	7.737772	16.98036	6.35756	-1.493254221	1.506627894	0.000184094	0.00013603
ENSRNOG00000025764	AC128848.1	4.90266	145.8231	17.10215	-5.276603343	3.197798212	9.70E-14	6.80E-07
ENSRNOG00000025889	Gnas	0.049984	1.950771	0.351614	-5.615963667	2.495772812	0.00229466	0.001053268
ENSRNOG00000026235	Hk3	0	3.269474	0.043378	-14.35393895	6.272273054	4.19E-15	1.32E-07
ENSRNOG00000026293	Jun	15.38989	74.16426	22.45585	-2.627969371	1.815310211	2.07E-07	0.000145288
ENSRNOG00000026647	Cxcl16	1.884715	28.01064	5.155461	-4.255166217	2.545387827	6.79E-09	0.000132331
ENSRNOG00000027024	Rgs16	2.545694	10.52784	2.77818	-2.396463248	2.008527074	7.98E-05	0.000606198
ENSRNOG00000027655	Clec4b2	0	0.083845	0	-10.05829678	10.21636278	0.001441042	0.000992228
ENSRNOG00000027855	Fcar	0	0.114132	0	-10.95499966	11.11312933	0.000506076	0.000310074
ENSRNOG00000028043	Cxcl3	0	2.492454	0	-13.7778087	13.93600745	5.63E-07	3.59E-07
ENSRNOG00000028801	Gsap	0.228209	1.672304	0.339159	-3.237094277	2.394990847	2.17E-07	2.56E-05
ENSRNOG00000028872	Rai14	2.259399	6.383142	2.145981	-2.073832808	1.702033602	2.42E-05	0.000666831
ENSRNOG00000028896	A2m	0.978109	6.0023	1.631977	-2.964679192	1.978201486	2.03E-11	0.000326074
ENSRNOG00000029662	Wdfy4	0.322388	1.185388	0.393349	-2.232175297	1.681646105	3.69E-08	4.88E-05
ENSRNOG00000029663	Il1rapl1	23.77588	4.759091	9.257577	2.497280161	-1.256356005	1.29E-17	0.000208603
ENSRNOG00000029682	Clic1	4.956499	73.36555	13.11167	-4.251682577	2.579894083	2.62E-15	1.50E-07
ENSRNOG00000030118	Msn	7.291458	48.70567	8.978667	-3.134467681	2.589885267	4.57E-11	3.02E-08
ENSRNOG00000030187	Mmp12	0.024765	0.470023	0	-4.538090687	11.41225541	0.004933893	1.07E-07
ENSRNOG00000030269	Atp2b2	169.1962	33.98462	82.94539	1.983455887	-1.222938581	7.17E-07	0.000978143
ENSRNOG00000030387	Kng1	0	20.42917	1.619589	-17.11614604	3.768105278	1.52E-30	6.55E-05
ENSRNOG00000030712	RT1-A2	5.198186	23.88233	4.036674	-2.450930071	2.292879075	8.30E-06	7.59E-06
ENSRNOG00000031167	AABR07054319.1	11.01173	40.2109	7.899016	-2.231660037	2.443980591	1.54E-05	1.98E-06
ENSRNOG00000031312	Tnfrsf1a	4.18839	21.04828	7.312869	-2.686457504	1.614403496	1.11E-08	0.000231176
ENSRNOG00000031743	Gbp2	0.958116	10.18079	1.568946	-3.769070372	2.788108146	2.35E-12	2.30E-08
ENSRNOG00000032436	Tmod3	7.163538	20.98177	9.685147	-1.904248017	1.199028851	6.59E-08	0.000652232
ENSRNOG00000032788	Dysf	0.137615	0.707272	0.174636	-2.717584335	2.117141486	1.15E-05	0.000242962
ENSRNOG00000033134	Mef2c	98.9244	31.81715	73.14643	1.597317627	-1.391683453	0.000242092	5.18E-05
ENSRNOG00000033173	Fam71e2	0	0.096428	0	-9.602957218	9.760968392	0.000379316	0.000219972

ENSRNOG00000033192	Osmr	0.694789	5.478572	0.437317	-3.343622259	3.748508476	1.58E-06	1.39E-07
ENSRNOG00000033256	LOC691141	0.286216	2.320983	0.222973	-3.352856974	3.550909615	0.000437102	2.81E-05
ENSRNOG00000033433	Csrnp1	2.692824	19.25093	2.513694	-3.205483602	3.038348511	3.93E-06	8.30E-06
ENSRNOG00000033527	Pappa1	0.036646	0.792187	0.058441	-4.779058116	3.823764589	7.49E-07	1.14E-06
ENSRNOG00000033528	Tll1	0.793498	3.297938	1.022015	-2.418851583	1.781515906	4.52E-07	0.000130323
ENSRNOG00000033570	Arhgap8	0.133604	1.730837	0.210559	-4.063742116	3.131743296	8.90E-08	8.56E-06
ENSRNOG00000036689	P4hb	38.32118	94.03623	38.99733	-1.652561834	1.358449754	8.13E-06	0.000198433
ENSRNOG00000036703	Itgax	0.068111	2.151548	0.089413	-5.327677706	4.693489687	6.24E-06	8.64E-06
ENSRNOG00000037113	Slfn2	0.744691	8.556132	1.626527	-3.911342089	2.494545004	7.02E-07	0.000406361
ENSRNOG00000037409	Scimp	0.010122	5.959309	0.084035	-9.396614384	6.312915996	1.66E-07	4.73E-08
ENSRNOG00000037865	Hormad2	0.012353	0.326052	0.020423	-5.083008198	4.080702817	0.007639034	0.000311092
ENSRNOG00000038047	Mt1	2.469941	72.60313	7.835678	-5.207893168	3.31355947	3.46E-07	0.000265568
ENSRNOG00000038132	Vsig4	0	0.612401	0.011591	-13.21148654	5.80791095	5.18E-09	0.000508843
ENSRNOG00000038722	Tlr1	0.110653	1.836326	0.195178	-4.410261754	3.321453676	4.81E-07	3.86E-05
ENSRNOG00000038835	Cd86	0.336591	4.114932	0.713988	-4.095714551	2.540506718	1.64E-10	1.17E-05
ENSRNOG00000038881	Hcls1	0.948257	10.70062	2.654881	-3.853782969	2.097428589	6.46E-16	4.25E-07
ENSRNOG00000038894	AABR07072096.1	0	0.067753	0	-10.27327506	10.43136046	0.001071286	0.00073415
ENSRNOG00000039197	LOC108348074	0.178517	1.074338	0.085739	-2.958935189	3.7397238	0.000144612	1.30E-05
ENSRNOG00000039464	Tmem229a	13.58933	1.225353	6.009898	3.107926556	-2.201104507	1.43E-07	0.000320978
ENSRNOG00000039744	RT1-CE4	5.54775	21.79389	4.797531	-2.577781045	2.472390623	1.10E-06	5.35E-06
ENSRNOG00000039902	Lbh	9.662174	33.75073	13.49507	-2.165580231	1.408153009	7.71E-07	0.000896847
ENSRNOG00000040287	Cyp1b1	1.120726	31.6127	6.625007	-4.950826125	2.426468875	1.40E-10	0.000228542
ENSRNOG00000042080	Il31ra	0	0.05237	0	-10.62103125	10.7791468	0.000979541	0.000651516
ENSRNOG00000042838	Junb	19.20289	90.67205	21.66985	-2.607685118	2.162285588	5.83E-05	0.000564091
ENSRNOG00000043044	Cnn2	3.519062	26.77889	6.410974	-3.28839609	2.159160146	4.55E-08	0.000111392
ENSRNOG00000043098	Mt2A	45.27849	887.2805	165.8694	-4.654842549	2.515980831	1.44E-20	1.85E-07
ENSRNOG00000043099	Ddx21	8.45446	16.55246	7.06283	-1.328121893	1.315867451	0.001075111	0.000982369
ENSRNOG00000043416	Bcl3	0.081795	4.656189	0.340365	-6.17603881	3.879041508	3.58E-11	2.55E-06
ENSRNOG00000043451	Spp1	0.940327	159.0937	5.296464	-7.771252652	5.034327324	3.11E-13	1.02E-07
ENSRNOG00000045654	LOC108348108	0.59121	87.79134	1.742069	-7.511401276	5.747110217	1.88E-26	8.52E-21
ENSRNOG00000045772	LOC100911545	0.79885	6.798044	1.843166	-3.429215443	1.975548119	5.75E-07	0.000323171
ENSRNOG00000046327	Rbpj	11.35128	18.25731	7.680785	-1.046949454	1.338308951	0.008006152	0.000833942
ENSRNOG00000046452	Fcgr2b	0.867572	25.72374	3.666424	-4.840600126	2.800781058	8.25E-13	4.96E-08

ENSRNOG00000046667	Fosb	2.785585	20.05339	1.986194	-3.221953685	3.417780061	8.64E-05	3.58E-05
ENSRNOG00000046699	Slpi	0	13.97157	0.086192	-14.15031752	7.369452535	4.13E-11	2.17E-07
ENSRNOG00000046834	C3	1.720631	7.617387	2.662066	-2.476234244	1.593484729	4.65E-08	0.000334376
ENSRNOG00000047606	Bcl2a1	0.673481	8.84699	1.544205	-4.091547246	2.619502348	3.35E-06	0.000368693
ENSRNOG00000048273	Apod	58.30635	187.5799	86.43906	-2.047776937	1.204697828	8.22E-08	0.000739499
ENSRNOG00000048771	RGD1559482	0.141085	6.349358	0.520711	-5.713185597	3.653721918	1.24E-08	4.69E-05
ENSRNOG00000049075	Fabp5	12.26215	92.85364	29.6537	-3.281209182	1.739342007	3.68E-12	6.54E-05
ENSRNOG00000049236	Myh9l1	7.877046	23.37855	10.31366	-1.924868953	1.267848929	4.58E-08	0.000240075
ENSRNOG00000049491	RT1-DMb	2.930397	18.01454	5.16009	-3.050476278	1.885966942	3.42E-07	0.000226948
ENSRNOG00000049517	Tnfaip3	2.452905	9.647608	2.14648	-2.340498265	2.265679082	0.000843695	0.00095549
ENSRNOG00000049700	LOC100909857	0	0.594849	0	-12.89113522	13.04932428	1.62E-10	3.10E-11
ENSRNOG00000049723	LOC102547963	0.054879	1.00305	0.029755	-5.787881827	6.404724609	6.61E-08	1.48E-05
ENSRNOG00000049895	LOC100910143	1.571436	0	0.626161	14.14802053	-13.26458184	1.43E-05	0.000334266
ENSRNOG00000050024	Ms4a4a	0.428787	5.463753	1.678145	-3.989249963	1.810738095	6.61E-10	0.000919342
ENSRNOG00000050183	RT1-CE1	0.005913	0.277647	0.008555	-6.491957645	5.380784148	0.000920541	0.000549545
ENSRNOG00000050190	Eng	2.929166	14.38062	2.659953	-2.659999369	2.53727147	0.000401662	0.000511808
ENSRNOG00000050251	MGC105649	0.045701	4.084249	0.148366	-6.726105342	4.877313572	4.46E-06	0.000127262
ENSRNOG00000050404	Pmepa1	9.483079	41.38459	11.68179	-2.488543662	1.917308493	2.14E-06	0.000181562
ENSRNOG00000050430	Vav1	0.796524	4.434298	1.656272	-2.73836424	1.901456776	3.09E-06	0.000660257
ENSRNOG00000050473	Rps27l	6.156573	40.06076	12.12936	-3.065361963	1.812026853	7.85E-09	8.84E-05
ENSRNOG00000050647	Hspa1b	0.621388	83.67021	1.648656	-7.419896118	5.756239871	4.63E-29	5.85E-23
ENSRNOG00000050697	Ctsz	5.71596	61.05381	13.95718	-3.776891068	2.220093224	2.60E-18	1.74E-08
ENSRNOG00000050794	Pdlim4	13.13989	65.97019	27.51494	-2.680593335	1.344507084	4.98E-15	9.70E-06
ENSRNOG00000050869	Cebpd	3.419129	47.91339	4.786426	-4.161495821	3.419156016	2.15E-07	5.78E-06
ENSRNOG00000050997	Ifrd1	21.82406	50.86547	17.02076	-1.582097256	1.671670867	0.000123631	4.65E-05
ENSRNOG00000051534	AC132720.2	0.010407	3.727865	0.304107	-8.522318747	3.692032289	7.37E-07	0.000696152
ENSRNOG00000051624	Hspe1	6.782828	22.67628	10.92427	-2.081391659	1.139883701	9.64E-09	0.000600859
ENSRNOG00000052015	AABR07042937.1	11.52213	0	4.999251	13.31194005	-12.5121214	1.52E-06	9.94E-06
ENSRNOG00000052038	Haus8	1.185192	11.49673	2.074776	-3.626879786	2.570910157	2.02E-07	5.46E-05
ENSRNOG00000052064	Parvg	1.352046	4.72899	1.444187	-2.163437728	1.799682058	0.000233388	0.001053906
ENSRNOG00000053272	Chi3l1	18.33767	80.91339	32.17635	-2.494283201	1.416854242	2.57E-12	2.48E-05
ENSRNOG00000053550	Itga1	2.300609	6.155681	2.027516	-1.710758137	1.675803022	0.000505978	0.001044163
ENSRNOG00000054251	Clec7a	0.085588	4.914524	0.312783	-5.774352254	3.960156457	3.13E-07	2.04E-05

ENSRNOG00000054336	Majin	2.79439	0.053571	1.954893	5.90818545	-5.665259921	1.22E-06	7.82E-05
ENSRNOG00000054860	Clec12a	0.234731	8.556716	1.135528	-4.894149615	2.804377105	8.86E-08	1.87E-05
ENSRNOG00000054890	Flna	6.36798	24.56351	8.745198	-2.30645076	1.58100051	4.88E-08	0.000168989
ENSRNOG00000055512	Hist1h2bk	4.407655	36.80677	12.26789	-3.41692079	1.660564179	1.72E-08	0.000972725
ENSRNOG00000055672	Gpx2	0.107582	18.28133	1.516168	-7.724716304	3.692438828	3.07E-08	1.35E-05
ENSRNOG00000056219	Olr1	0.322753	4.01472	0.196003	-4.00910948	4.451848244	1.67E-06	8.06E-08
ENSRNOG00000056786	Piezo1	1.197411	4.720097	1.164073	-2.337641133	2.10884665	8.84E-07	9.91E-06
ENSRNOG00000057058	Cd300a	1.526278	5.024736	1.789528	-2.080408454	1.581161005	4.95E-07	2.95E-05
ENSRNOG00000057153	Pla1a	1.075494	8.159561	0.950447	-3.284068904	3.199152853	2.37E-07	1.83E-06
ENSRNOG00000057315	Kcnh3	35.2072	9.17961	23.73249	1.625264493	-1.320596455	1.31E-05	0.000260102
ENSRNOG00000057451	Itga5	0.551813	5.938237	0.660906	-3.792311655	3.261709342	5.28E-07	7.48E-06
ENSRNOG00000057832	Rnf125	0.28451	1.780322	0.277211	-3.002544261	2.779282032	0.00015133	0.000161433
ENSRNOG00000058111	Itga2	0	0.429336	0	-14.4222076	14.58040992	8.15E-14	2.00E-14
ENSRNOG00000058186	Errfi1	14.46718	44.51974	11.16666	-1.943921564	2.076850284	0.000166679	2.42E-05
ENSRNOG00000058388	Zfp36	2.323521	28.91342	3.30928	-4.006913109	3.222908982	2.13E-06	4.48E-05
ENSRNOG00000058568	Dhrs9	0.495239	7.921897	0.679257	-4.356924007	3.644219515	9.22E-08	6.34E-06
ENSRNOG00000058645	Tnc	2.912365	8.330635	2.288058	-1.88356621	1.960769374	0.000447606	0.00022377
ENSRNOG00000058697	LOC103690319	0	0.140089	0	-8.292967285	8.450696044	0.001347545	0.000975786
ENSRNOG00000059381	Mrps6	12.41603	60.54116	25.32223	-2.634185845	1.341706757	6.68E-12	0.000162375
ENSRNOG00000059461	B4galt1	1.275067	6.013722	2.142111	-2.592378661	1.576792966	1.58E-08	0.000422052
ENSRNOG00000059463	Slc39a1	18.96004	61.18251	25.75459	-2.043248984	1.332584373	6.98E-08	0.000211383
ENSRNOG00000059947	Sdc1	0.325687	9.789851	0.61786	-5.277154114	4.086692221	4.08E-09	3.12E-07
ENSRNOG00000060338	Lipc	0	0.025304	0	-10.67663626	10.83475299	0.000534539	0.00034017
ENSRNOG00000060381	Col15a1	0.26772	1.075742	0.226933	-2.366151607	2.337283376	0.000447622	8.65E-05
ENSRNOG00000061595	Tfec	0.01856	0.992863	0.136501	-6.066412075	2.955944505	0.000559717	1.20E-05
ENSRNOG00000061857	Mgst2	0.862539	8.955455	1.996012	-3.73618403	2.256662095	2.86E-08	0.000477124
XLOC_057615	XLOC_057615	12.965	0.602261	2.621154	4.287052498	-2.327389119	3.93E-16	0.00046927
XLOC_145157	XLOC_145157	9.551363	18.50738	6.366644	-1.302084574	1.617359348	0.002737782	0.000269032

Table S3. Differentially expressed miRNAs associated with the effect of 20(*R*)-ginsenoside Rg3 on stroke recovery.

miRNA_ID	Sham	Model	Rg3	log2FoldChange		Pvalue	
				Sham_vs_Model	Model_vs_Rg3	Sham_vs_Model	Model_vs_Rg3
novel_62	769.4574	4538.814185	727.5542	-2.56023	3.042989	0.002637388	6.32E-08
rno-let-7g-3p	11.85656	97.7889871	9.366126	-3.02734	3.80309	6.00E-05	2.11E-06
rno-miR-128-2-5p	232.1279	52.94668035	143.6822	2.097066	-1.05391	2.44E-07	0.024106
rno-miR-130a-5p	3.910058	26.7645146	13.3146	-2.72846	1.384491	0.001033366	0.043599
rno-miR-130b-5p	27.15453	11.88339705	40.27568	1.170238	-1.37061	0.041246653	0.014911
rno-miR-142-3p	6.100142	186.5200239	54.87105	-4.92151	2.167438	1.85E-15	0.001033
rno-miR-147	1.144834	13.08606324	2.216147	-3.47744	2.922856	0.001772933	0.00168
rno-miR-155-5p	9.550528	79.04928925	35.08951	-3.06028	1.552389	1.64E-07	0.001951
rno-miR-1949	0.645657	10.66992636	0.689063	-4.08423	4.33143	0.009855444	0.001932
rno-miR-19a-3p	6.33792	26.20781728	13.35822	-2.04448	1.364118	0.003185878	0.020989
rno-miR-204-3p	66.56293	9.866032793	33.87694	2.666021	-1.35104	9.81E-07	0.036346
rno-miR-21-3p	0	19.50004994	2.555326	-6.87379	3.364795	1.12E-05	0.002454
rno-miR-21-5p	23588.52	255201.8563	149404.2	-3.43549	1.15347	6.75E-60	1.16E-05
rno-miR-223-3p	10.90986	69.8025031	41.82603	-2.66428	1.120901	4.65E-05	0.040755
rno-miR-301a-3p	10.13306	51.54964243	21.98678	-2.32817	1.620959	9.92E-06	0.002977
rno-miR-337-3p	3.85419	34.10199951	18.15699	-3.13404	1.286058	1.08E-05	0.026014
rno-miR-3590-3p	3.910058	26.7645146	13.3146	-2.72846	1.384491	0.001033366	0.043599
rno-miR-362-3p	5.450961	73.46944012	39.16325	-3.72272	1.301165	2.02E-09	0.030373
rno-miR-449a-5p	7.372047	29.32395453	6.643843	-1.98333	2.522148	0.023687632	0.001061
rno-miR-503-5p	32.05873	119.0095406	71.15599	-1.88901	1.131556	0.00022377	0.016252
rno-miR-543-3p	5856.427	2026.355905	5579.826	1.53011	-1.1056	0.000975786	0.011269
rno-miR-671	657.7029	126.1747237	344.4962	2.367292	-1.08035	0.000162375	0.01763
